# Treatment Strategies for Dilated Cardiomyopathy in Children: Scientific Statement from the American Heart Association—A Real Advance! But Please More Specific!

**DOI:** 10.1007/s00246-023-03314-7

**Published:** 2024-02-03

**Authors:** Dietmar Schranz

**Affiliations:** Kinderkardiologie, Universitätskinderklinik, Goethe Universität Frankfurt, Theodor-Stern-Kai 7, 60590 Frankfurt, Germany

The American Heart Association's (AHA) insightful scientific statements on treatment strategies for dilated cardiomyopathy (DCM) in children are a real advance but have led me to make some additional and probably necessary comments, suggestions and corrections.

Influenced by Karl Popper’s writings “*on the so-called sources of human knowledge*” [1], which describe, among other things: “*an authoritative attitude towards problems of human knowledge exists when assertions are only permissible if we rely on the authority from sources of knowledge*.” Transferred to medicine, it seems to be congruent with the “authority” of guidelines. However, according to Popper, “*there is no such “authority”, so all that remains is a critical debate. Scientific theory should make predictions that can be tested, and the theory should be discarded when those predictions are found to be wrong. All forms of epistemological fundamentalism have to be rejected. Science advances when it uses deductive reasoning as its primary focus*.” What do Popper's thoughts mean when applied to the conceptual discrepancies in the treatment of heart failure (HF) in children? Treatment strategies to achieve regeneration versus symptom-oriented therapy with accelerated cardiac degeneration and, as a last resort, heart transplantation (HTX). There is only one randomized, i.e. “authorized” study on ß-blockers (BB) in children [2]. This study showed no improvement in outcome and symptoms by adding carvedilol to ACE-inhibitors. Bogle et al. have re-cited this study again [3], as it has been conducted more than 500 times over the past nearly two decades with the purported claim that “BBs don’t work in children with heart failure”. “*Since science is a critical activity, it makes it obligatory to combat dogmatic thinking by critically reviewing one’s own hypotheses a duty*”. So, what supports the hypothesis that BBs in general, and ß1-selective beta-adrenergic blockers in particular are effective in infants with DCM? That it is therefore not questionable, but requires a differentiated recommendation?

Again, according to Popper “*criticize the hypotheses to find errors and get closer to the truth by satisfying three requirements*”:

1. *The new hypothesis must refute the old thesis*: ß-blockers, regardless of whether one has to be used with a non-specific or a highly selective ß1-adrenoceptor blocker profile. An elevated heart rate in pediatric heart failure can easily be lowered by more than 30% of baseline values [4]. A threshold of efficiency by simply lowering heart rate is easily measurable for everyone, including the parents. Clinical improvement, based on prolonged coronary and transpulmonary perfusion time, ventricular-filling time and improved ratio of myocardial and total body oxygen supply to O2 consumption is monitorable by pathophysiological imaging data, but also from clinical symptoms such as respiratory rate, eating behavior and weight gain.
2. *The new hypothesis avoids some flaws of the previous hypothesis*: The previous hypothesis, that ß-blockers are ineffective in pediatric heart failure because of the differences from adult HF, was again based on the cited study [2]. However, the “guideline-relevant”, randomized, double-blind study protocol did not take into account the inclusion criteria of the patients examined [2]. It is obvious that the patient’s specifications did not allow any evidence of the effectiveness of a drug, particularly not the non-specific ß-blocker [5]. Patient heterogeneity combined with the low severity of heart failure, with mean baseline BNP-values of 91 pg/ml and heart rates in placebo- and carvedilol-treated patients of 106 bpm of 90 bpm, respectively, did not provide an answer to the nature of the HF action by BB. Baseline values were already consistent with the ultimate treatment goal when severe or even end-stage pediatric heart failure is treated with BB [6].
3. *The new hypothesis can explain things that the old hypothesis cannot explain or predict*: Infants with left ventricular DCM in whom HTX is or should be considered nonetheless have a high chance (> 70%) for functional regeneration despite such an advanced stage [7]. However, this depends on both (a) new strategies to promote ventriculo–ventricular interaction, such as pulmonary artery banding (PAB), that the authors Bogle et al. have generously taken into account in the “future perspectives”, and (b) on medical strategies that promote regeneration instead of cardiac degeneration. Beta1-adrenoceptor blockers play a central role, especially in young children with DCM in whom ß-2 adrenergic receptors are already downregulated [5]. In this context, the lower recovery rate of only one-third in the US PAB-trial versus more than two thirds in Germany was not due to a selection bias as such due to a sicker patient population, but was probably based on a completely different perioperative treatment concept [8]. On which I, partly involved in the US-PAB study, tried to have more influence. The ß1-specific adreno-receptor blocker can be used in sufficient doses which, as mentioned, can be easily monitored by the decrease in heart rate or other hemodynamic parameters. This, almost without side effects, especially without ß2-receptor-blocker-related bronchoconstriction. Basic research and clinical data suggest that a ß1-blocker does more than just block ß1-receptors with antiarrhythmic properties. Beta1-adrenoreceptor blockade has been shown to prevent myocardial apoptosis and necrosis, restore ischemia-induced down-regulated excitation-contracting proteins and mitochondrial function, and further stimulate cardiac progenitor cell survival and proliferation. In addition, cardioprotective cross-signals are to be expected through residual ß2- and ß3-related mechanisms [9]. The targeted use of ß1-receptor blockers in young DCM patients, especially with advanced heart failure, is overdue [10].
